# Machine learning models predicting multidrug resistant urinary tract infections using “*DsaaS*”

**DOI:** 10.1186/s12859-020-03566-7

**Published:** 2020-08-21

**Authors:** Alessio Mancini, Leonardo Vito, Elisa Marcelli, Marco Piangerelli, Renato De Leone, Sandra Pucciarelli, Emanuela Merelli

**Affiliations:** 1 School of Biosciences and Veterinary Medicine, University of Camerino, Camerino, Italy; 2 ASUR Marche AV2, Operative Unit of Clinical Pathology, Senigallia, Italy; 3 School of Science and Technology, Computer Science Division, University of Camerino, Camerino, Italy; 4 School of Science and Technology, Mathematics Division, University of Camerino, Camerino, Italy

**Keywords:** Machine learning, Classification, Regression, Data science pipeline, Antibiotic stewardship, Multi drug resistance, Nosocomial infection

## Abstract

**Background:**

The scope of this work is to build a Machine Learning model able to predict patients risk to contract a multidrug resistant urinary tract infection (MDR UTI) after hospitalization. To achieve this goal, we used different popular Machine Learning tools. Moreover, we integrated an easy-to-use cloud platform, called *DSaaS* (*Data Science as a Service*), well suited for hospital structures, where healthcare operators might not have specific competences in using programming languages but still, they do need to analyze data as a continuous process. Moreover, *DSaaS* allows the validation of data analysis models based on supervised Machine Learning regression and classification algorithms.

**Results:**

We used *DSaaS* on a real antibiotic stewardship dataset to make predictions about antibiotic resistance in the Clinical Pathology Operative Unit of the Principe di Piemonte Hospital in Senigallia, Marche, Italy. Data related to a total of 1486 hospitalized patients with nosocomial urinary tract infection (UTI). Sex, age, age class, ward and time period, were used to predict the onset of a MDR UTI. Machine Learning methods such as Catboost, Support Vector Machine and Neural Networks were utilized to build predictive models. Among the performance evaluators, already implemented in *DSaaS,* we used accuracy (ACC), area under receiver operating characteristic curve (AUC-ROC), area under Precision-Recall curve (AUC-PRC), F1 score, sensitivity (SEN), specificity and Matthews correlation coefficient (MCC). Catboost exhibited the best predictive results (MCC 0.909; SEN 0.904; F1 score 0.809; AUC-PRC 0.853, AUC-ROC 0.739; ACC 0.717) with the highest value in every metric.

**Conclusions:**

the predictive model built with *DSaaS* may serve as a useful support tool for physicians treating hospitalized patients with a high risk to acquire MDR UTIs. We obtained these results using only five easy and fast predictors accessible for each patient hospitalization. In future, *DSaaS* will be enriched with more features like unsupervised Machine Learning techniques, streaming data analysis, distributed calculation and big data storage and management to allow researchers to perform a complete data analysis pipeline. The *DSaaS* prototype is available as a demo at the following address: https://dsaas-demo.shinyapps.io/Server/

## Background

Nowadays, healthcare operators must process and interpret large amounts of complex data [1]. Machine Learning methods have broadly begun to infiltrate the clinical literature in such a way that the correct use of algorithms and tools can facilitate both diagnosis and therapies. The availability of large quantities of high-quality data could lead to an improved understanding of risk factors in community and healthcare-acquired infections. For instance, in the antibiotic stewardship field, researchers utilized Massachusetts statewide antibiogram data to predict three future years of antibiotic susceptibilities using Machine Learning regression-based strategies [2]. To date, international guidelines recommend to use institutional antibiograms in the development of empiric antibiotic therapies [3] and Machine Learning methods could help physicians in the empirical treatment of the urinary tract infections (UTIs). These are usually known as the most common bacterial infections with a significant financial burden on society [4]. In hospitals at least 40% of all infections are UTIs and bacteriuria develops in up to 25% of patients who require a urinary catheter for 1 week or more [5]. The selection of adequate treatment for the management of UTIs is increasingly challenging due to their etiology, bacterial resistance profile, and evolving of adaptive strategies. Moreover, the bacteria resistance to antibiotics has risen dramatically with increasingly fewer therapeutic options. One of the causes is the recurrent infection that leads to development of multidrug resistance (MDR). Several risk factors are associated with UTIs, including sex and age [6]. Male patients have a lower risk of contracting uncomplicated UTIs but more prominent to contract complicated or MDR infections than women. Older adults are more prone than younger individuals in developing urinary tract infections because of incomplete bladder emptying (often related to prostatic enlargement in men), higher rate of catheter usage and increased susceptibility to infection associated with frailty [7]. Moreover, infections caused by MDR organisms are more common in elderly people, especially those with catheters or residing in long-term care. The resistance rates to antimicrobials in UTIs can differ from region to region, patient to patient and even from ward to ward where the patient is hospitalized. Hence, in a nosocomial infection it is important to know the microorganism population in the hospitalization place [8].

Unfortunately, antibiotics are not always prescribed responsibly contributing to the development of new resistances [9]. To effectively treat patients and prevent the increases in resistance, every institution must have an up-to-date susceptibility knowledge. Moreover, predictions can be used to guide prescription practices and prepare for future resistance threats [2].

The objective of this work is to design, develop and evaluate with a real antibiotic stewardship dataset, a predictive model useful to predict MDR UTIs onset after patient hospitalization. For this purpose, we implemented an online, completely dynamic platform called *DSaaS* and specifically designed for healthcare operators to train predictive models (supervised learning algorithms) to be applied in this field.

## Methods

In this section we describe both the methods used for the engineering and implementation of the platform (*DSaaS)* and for the analysis of the real database about antibiotic stewardship.

### DSaaS platform

*DSaaS* is an online, user-friendly, platform that allows us to validate simple predictive models based on regression algorithms as well as to utilize supervised classification techniques. *DSaaS* allows to manage in an easy way the whole pipeline required for extracting some knowledge from data: data preparation/ data pre-processing, data analysis, applying predictive models and data visualization. The *DSaaS*’s tools used to build the pipeline are reported below in a chronological order:

- Data injection: *DSaaS* uses Apache Nifi for data extraction, routing and transformation;
- Data engineering: *DSaaS* allowed us to carry on data type transformation, data filtering, data selection, under and oversampling [10] and gave us the possibility to transform categorical variables into a series of dichotomous variables, namely variables that can have a value of zero or one only, by using a dummy variables approach;
- Three different supervised Machine Learning classification algorithms: *Support Vector Machines*, one of the most widely used classification method, specifically designed for binary classification problems looking for a hyperplane with maximum margin of separation between the two classes [11]; *Catboost*, a recently implemented algorithm especially suitable for categorical datasets [12] and *Neural Networks*, a very well-known technique aiming to reproduce the behavior of human brain where neural cells (i.e., neurons) receive, process and transmit external data with each other [13, 14].
- Auto ML: *DSaaS* provides an auto Machine Learning approach that allows the user to identify the best parameter input values for the chosen Machine Learning methods [15];
- Validation and Cross-Validation: in the context of predictive modeling, when comparing supervised classification models, *DSaaS* provides different indexes. In our experiment we used accuracy (ACC), area under receiver operating characteristic curve (AUC-ROC), area under Precision-Recall curve (AUC-PRC), F1 score, sensitivity (SEN), specificity and Matthews correlation coefficient (MCC). At the moment *DSaaS* allows the user to apply k-fold Cross-validation and to use a number of folds that is automatically determined by *DSaaS* taking into account the size of the data frame. Details of all evaluators available in *DSaaS* can be found in the supplementary material;
- Data Visualization: for every Machine Learning algorithm *DSaaS* provides visualization tools that allow the user to understands the resulting model.

We provide a complete list of *DSaaS* functionalities in the Supplementary Materials.

*DSaaS* in future will allow to create and execute more complex data analysis processes: an easy dataflow editor will permit to publish the results obtained as a REST service [16]. Moreover, *DSaaS* will allow to use scripting for exploiting the power of R language. Lastly, we are also planning to provide *DSaaS* with a “Stewardship UI” that will help users to maintaining data quality within *DSaaS* platform [17].

### Dataset

The dataset was built out based on the bacterial isolates reports of the Clinical Pathology Operative Unit of the Principe di Piemonte Hospital in Senigallia, Marche, Italy. The hospital has 288 beds and a mean of 31,000 inpatient days per semester. We included in the study all patients admitted from March 2012 to March 2019 (7 years). Only isolates, collected from infections that occurred 48 h after admission, were used and identified as nosocomial infections, as defined by the Centers for Disease Control and Prevention (CDC) [18].

We considered as MDR UTI a patient with a microorganism resistant to one or more antibiotic classes as defined from the CDC [19] and we assigned the value R (R = 1) to all MDR UTI and the value S (S = 0) to the rest.

We collected results from 11 wards, spatial units provided with rooms where a unique staff of health-care and co-workers are active. In our model we considered the variable “ward” as a space subjected to few interactions with the others. Therefore, the microbial population within a ward with their related hospital infections and antibiotic resistance profiles were preserved for each ward and time period.

To test the *DSaaS* platform we decided to restrict the database and to use only the urine samples, corresponding to the most commonly requested clinical test among wards. The selection of five predictors (time-period, sex, age, age class and ward) was primarily based on urinary tract infection related literature [20]. Table 1 shows the detailed operational definition of variables used in our study. A total of 1486 clinical samples were considered for this study. Specimens were processed according to good laboratory practice and standard methods for identification.

**Table 1:** Operational definition of variables

Variables		Measurements	Definition
Dependent	MDR Resistance	Discrete	Does the patient acquire a MDR infection during hospitalization? Yes or No
Independent	Sex	Discrete	Sex of the patients, Male or Female.
	Age	Continous	Age (in years) during hospitalization
	Age Class	Discrete	10 years class to witch the patient belong, from 1 to 10
	Ward	Discrete	Ward where the patient was hospitalized, from 1 to 11
	Time Period	Discrete	Time period in which the patient was hospitalized in a ward, from 1 to 14

### Data pre-processing and data preparation

Duplicate data were discarded using the Bio-Mérieux VIGIguard™ software if all the following conditions were true: isolate collected from the same patient, same specimen, same ward, same species and similar antibiotic pattern (S/R = 1; I/R–S/I = 2) within 20 days.

*DSaaS* adopted the Caret v6.0–82 [13] and the GA (Genetic Algorithm optimization) v3.2 package [21] to automatically tune the optimal combinations of model parameters for the three Machine Learning algorithms we choose, aiming to achieve a better prediction performance. Evidence demonstrated that the class imbalance (unequal size of the classes), which is just the situation in our sample, can substantially impact the performance of the method used. Therefore, we adopted synthetic minority over-sampling technique by under-sampling the adequate class and over-sampling the inadequate class to improve the model performance [10]. *DSaaS* did also automatically a 10-fold cross validation method with three repeats, which has been viewed as the de facto standard for estimating model performance [22].

We randomly divided the database in a training set (70%), and a test set (30%) to evaluate the predictive models. The training set were used to build the classification algorithms using gradient boosting Catboost [12], Neural Networks (13–14) and SVM [10]. We refer to the Supplementary Material file for a detailed description of the algorithms.

### Performance measures

*DSaaS* allowed us to measure the model’s performance using accuracy, area under receiver operating characteristic curve, area under Precision-Recall curve, F1 score, sensitivity, specificity and Matthews correlation coefficient. To describe such performance measures for classification problem, it is essential to define a specific matrix, called confusion matrix, containing the number of false positives (FP), false negatives (FN), true positives (TP), and true negatives (TN). Specifically, a two-class (positive-negative) confusion matrix is a table where each row represents a predicted value and each column defines an actual value (or vice-versa): all correct prediction (TP and TN) are located along the matrix diagonal, while the errors are given by all the elements outside the diagonal.

Accuracy (ACC) [23] is a value that can be directly calculated from the confusion matrix and defines how often the classifier is correct and is calculated as the ratio between the number of correct predictions and the total number of predictions.

To define AUC [23] it is necessary to introduce the ROC curve (Receiver Operating Characteristic curve), namely a graph showing the performance of the classifier over all possible thresholds with respect to two parameters: the sensitivity (also known as recall or true positive rate, TPR) and the false positive rate (FPR). FPR is calculated as the ratio between the number of negative inputs wrongly classified as positive (false positive) and the total number of negative data and measures the proportion of all the negative inputs who will be identified as positive. AUC-ROC (Area Under the ROC Curve) measures the area underneath the ROC curve: it has a range of values from 0 to 1. The area measures discrimination, that is, the ability to correctly classify random positive and negative data.

Sensitivity [23] is calculated as the ratio between the number of positive inputs correctly classified as positive (true positives) and the total number of positive data, measuring how well the classifier made positive predictions based on all classes. It can be seen as the classifier ability to correctly detect positive inputs).

Specificity [23] also known as true negative rate (TNR), is defined as the ratio between the number of negative inputs correctly classified as negative (true negatives) and the total number of negative data. It measures how well the classifier made negative predictions based on all classes (it can be seen as the classifier ability to correctly detect negative inputs).

Matthews Correlation Coefficient (MCC) is a classification measure computed directly using the values of the confusion matrix: it takes values from − 1 to + 1, the first case describing a totally wrong classifier, the latter defining a faultless predictor.

F1 score is a weighted average of precision (ratio between true positives over the total number of positive elements) and sensitivity (namely, recall) values, therefore it is computed by adding the two values by a coefficient that defines its weight (importance) with respect to the other. F1 score has the best value at 1 and reaches the worst at 0.

Similarly to ROC curve, Precision Recall curve (PRC) plots precision and recall values for different thresholds: the value defining whether or not a data point is considered positive is continually changed and such results are graphically displayed. It is trivial to notice that, a high PRC value is linked to high recall and precision values.

Finally, overall model performance was calculated by averaging model performances over the time [23].

## Results

**Fig. 1:**
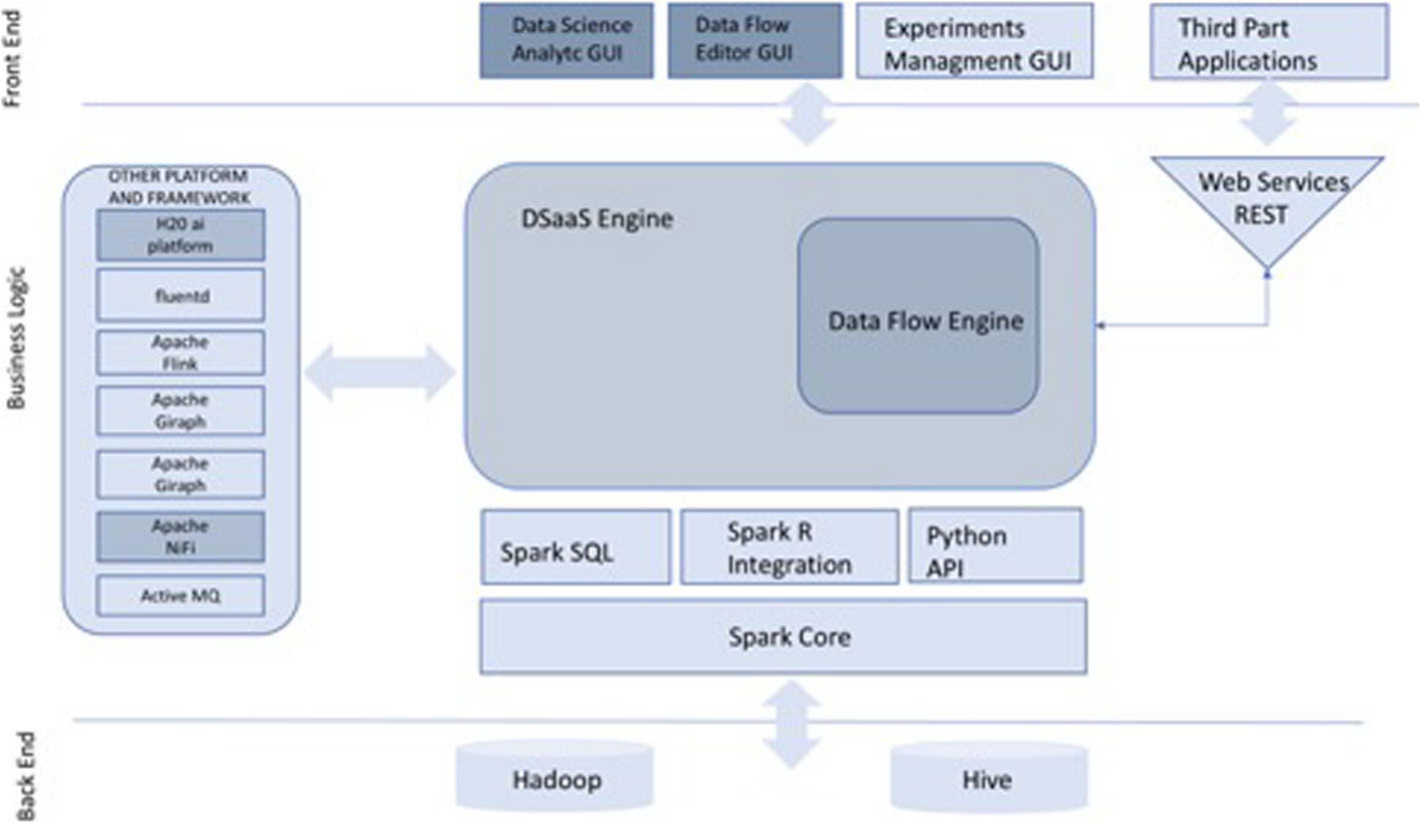
DSaaS future architecture. In dark gray are shown the operative modules described in this paper and already operative. In light gray are showed the modules that will be implemented in the future to perform data flow editing, R scripting and a Stewardship UI

To build the model, we used a dataset based on antibiotic resistance information obtained from a tertiary hospital in central Italy. After using different tools already present in literature, we decided to aggregate all we needed in a cloud platform called *DSaaS* that allows both testing of data analysis models and the creation of rough but useful Machine Learning processes easily usable by non-expert users. A demo version of *DSaaS* can be found at https://dsaas-demo.shinyapps.io/Server/ while its actual and future architecture is shown in Fig. 1*.*

Several supervised Machine Learning algorithms, readily available in the platform, have been used to predict the patient risk to acquire a MDR UTI and results were subsequently compared to obtain the best model possible to predict further resistance outcome.

Table 2 shows predictors and descriptive statistics for infected patients with and without an MDR urinary tract infection. Respectively 767 and 718 in-patients with and without hospital-acquired infections was present.

**Table 2:** Descriptive statistics for infected patients with/without an MDR urinary tract infection

Variable	Infected Patients with MDR UTIs	Infected Patients without MDR UTIs
	Summary statistics	
Sex	Male: 267, Female: 500	Male: 149, Female: 569
Age	M: 70,0 (SD 25,5)	M: 59.5 (SD 28.7)
Age Class		
Ward		
Time Period (six-months)		

Table 3 shows the results of the three Machine Learning algorithms we tested with *DSaaS*. Accuracy, AUC-ROC, sensitivity, specificity, MCC, F1 score and AUC-PRC were used to assess the performance of those methods. Since we adopted ten-fold cross validation for estimating model performance, the means and standard deviations of the above four metrics can be calculated for the training sample. Among the three methods employed, Catboost has the highest accuracy rate (0.711), followed by Neural Networks (0.646) and Support Vector Machine (SVM) (0.643) almost with the same performances. In terms of the AUC-ROC, Catboost (0.735) has a value higher than 0.7, indicating a more than acceptable classifier performance. The AUC-ROC values for Neural Networks and SVM are lower than 0.7, demonstrating poor performance. MCC, in the case of Catboost, is 0.909, resulting the best compared to NN and SVM. In these, the value of the MCC, however good, is 0.878 and 0.810 respectively. Both F1 score and AUC-PRC perform well in the case of Catboost and NNs, having results above 0.8.; SVM performs not very having values of 0.702 and 0.752 for the F1 score and AUC-PRC respectively.

**Table 3:** Performance evaluation of models using the test set. The standard deviation is reported in round brackets

Method	AUC-ROC	Accuracy	AUC-PRC	F1 score	MCC	Sensitivity	Specificity
Catboost	0.739 (0.021)	0.717 (0.032)	0.853 (0.028)	0.809 (0.027)	0.909 (0.026)	0.904 (0.061)	0.343 (0.052)
SVM	0.628 (0.025)	0.630 (0.057)	0.752 (0.031)	0.702 (0.033)	0.810 (0.032)	0.823 (0.042)	0.254 (0.085)
NeuralNetworks	0.652 (0.023)	0.686 (0.019)	0.801 (0.024)	0.804 (0.016)	0.878 (0.024)	0.880 (0.077)	0.288 (0.075)

Summing up, Catboost has a quite good performance while the remaining classifiers perform worse. Finally, the sensitivity (recall) value demonstrates us that all the three classifiers have a high performance in discriminating true positives (MDR infection) with values of 0.895, 0.807 and 0.752 respectively for Catboost, SVM and Neural Networks.

## Discussion

The field of bioinformatics is advancing from being a tool of pure statistical analysis and integration of data to a process able to support the definition of predicting models that evolve along with the biological experiments [24–27].

To date Machine Learning promises to assist clinicians in integrating ever-increasing loads of medical knowledge and patient data into routine care. Data-driven Machine Learning aims to identify patterns among multiple variables from huge data sets (big data) undiscoverable using traditional biostatistics [28] and Evidence Based Medicine (EBM) [29]. Traditional EBM shares these goals, but Machine Learning aims to achieve them more quickly and, since it uses data sets that are already available, Machine Learning has fewer constraints related to logistics, ethics, study design, and sample size than EBM [30]. Nowadays we are on the verge of a major shift in hospital epidemiology. Through the appropriate application of Machine Learning, healthcare epidemiologists will be able to better understand the underlying risk for acquisition of infectious diseases and transmission pathways, develop targeted interventions, and reduce hospital-acquired infections [30]. Machine Learning outperforms EBM and conventional statistic in many ways: algorithms can operate at the point of care using software embedded in investigational devices, electronic health records, or mobile device applications; during continuous monitoring predictive models can learn from new data; predictive models are flexible and an epidemiologist can incorporate more variables (antibiotics, bacteria, more medical facilities etc.) to refine his strategies in real time [28].

The design of a data analysis experiment may be deemed challenging because of the goal of the project not being known ex-ante. However, data analysis pipeline needs to be fast, efficient and correct, as well as easy and reproducible. Often, the goal is achieved by transforming original data and using different algorithms to identify the best prediction model. For this purpose, there are several tools that help data scientists to carry out their experiments. However, these tools are often difficult to use by a non-expert in programming language, for example a clinician. Some of these tools are development environments such as Bio7; others, such as Apache Zeppelin and Jupyter are collaborative platforms to manage and execute machine learning-oriented language scripts (for example python and R). Moreover, there are platforms allowing the specification of data analysis pipelines with workflow-oriented tools, such as Windows Azure. The one thing all these tools have in common is to be oriented data analysis experts with knowledge of programming languages. The aim here is to create a set of services that allows a domain expert user to perform and validate different Machine Learning methods over their data. Unlike the tools mentioned above, despite being characterized by less features, the *DSaaS* (Data Science as a Services) platform may be very useful for a domain expert aiming to use a Machine Learning algorithm on his data.

In our case-study, since we were dealing with data defined by binary targets describing whether an individual turned out to be affected by an MDR UTI or not, we decided to use a variety of well-known Machine Learning classification approaches previously implemented in *DSaaS*. In this way, we both studied the quality of the platform and were able to get a comparison of the classification performance using several classification models on the same dataset. Specifically, as a first step we decided to use Support Vector Machine (SVM), Neural Networks (NNs) and a quite new boosting method, known as Catboost. In particular, we decided to use SVM because of its manful advantages: SVM is a robust algorithm, very effective on slightly different datasets; it is safe, that is, it ensures the reduction of risks and uncertainties; it allows users to operate using kernels, transforming data from the original input set to a higher dimensional Hilbert space and, therefore, allowing non-linear classification. Neural Networks are now a classical tool, used for almost every Machine Learning experiment. Moreover, *DSaaS* allows the user to choose how many hidden layers and neurons to use, satisfying many different demands. Finally, we have chosen to use Catboost since it is particularly suitable for dataset with an important presence of categorical features, namely different from numerical ones that can only assume a limited, and usually fixed number of possible values corresponding to different types or categories. Moreover, Catboost uses a random forest structure, making the algorithm robust with a good ability to deal with training and test errors.

As target value we decided to assign 1 to individuals with the characteristic to have an MDR UTI (that means, R) to two or more antibiotic classes. Therefore, in our dataset the negatives coincided with non-MDR UTIs and are described by all the points with target value equal to 0 (that means, S). From Table 3 it can be noticed that, among the three algorithms the one having best results was Catboost: it achieved the best value in terms of sensitivity, AUC-ROC, accuracy rate and a very low value for specificity. Note that, specificity measure has low results in all three methods, while we have obtained generally fair results (that means, above 0,75) for sensitivity value. Having high value of sensitivity is very good for the model because it means that the type-II error is small; on the contrary having a low value of specificity means that the type-I error is high. Given that the best situation is when there are no errors, in this context is better to treat a person with a specific antibiotics even if he/she does not need it (type-I error) rather than not to treat at all even if he/she is going to develop a MDR infection (type-II error). By definition of sensitivity, we can conclude that our predictors have better results when a resistant data point (that means MDR), with target equal to 1, is considered. Hence, the used predictors have good performance indicating if a new hospitalized patient is at risk of taking a multi-drug resistant (MDR) infection. Furthermore, as regards SVM and Neural Networks, they have similar accuracy and AUC-ROC results around the value 0,6. Finally, the use of the MCC, F1-score and AUC-PRC gives us a better and complete overview of our model, even if those metrics are more informative and powerful if applied to unbalanced classes [31]. In particular, high values of the MCC indicates if that all the entries of the confusion matrix (true positive, true negatives, false positives and the false negatives) are very good in predicting the output of the model without overestimating the performance as, sometimes, happens using only accuracy and AUC-ROC [32]. In our case, Catboost outperforms NN and SVM in all the three metrics, resulting the best algorithm for the problem of MDR infection relating on our dataset.

Despite numerous studies have investigated risk factors in UTIs [18], literature revealed that little of those studies adopted Machine Learning techniques for prediction. At the best of our knowledge, our study is the first adopting a Machine Learning approach in predicting the patient-related risk after the hospitalization to acquire an MDR UTI. Then, by utilizing five different features easy to obtain from a new hospitalized patient, physicians may quickly adopt early prevention and intervention procedures and decision plans may be formulated in combination with related clinical experiences.

Furthermore, following the enhancement of the predictive model, the integration into the hospital computerized physician order entry could be done, where physicians may acquire a timely alert regarding the possibility of the onset of MDR UTIs in an early hospitalized patient.

Several limitations should be noted in our study. First, potential risk factors which were unavailable from the review of medical records are not considered here. In the next future we will point to enhance the model with other well-known UTIs risk factors like diabetes, the presence of a catheter, sexual-related factors, antibiotic use and renal transplantation [28]. Moreover, using Machine Learning techniques, risk factors commonly neglected by traditional statistical models can be discovered. Secondly, the analyzed cases were extracted from a small-scale hospital and therefore the generalizability of our findings may be limited. In the future we wish to gather more cases from a wider variety of hospitals.

## Conclusions

*DSaaS* can help physicians to construct easy and fast predictions models that could be helpful to treat hospitalized patients. Additionally, epidemiologists can use predictions to guide policies, research, and drug development for upcoming years. In the first version of *DSaaS,* we provided a useful prediction model for hospitalized patients on the onset of an MDR UTI with discrete performance. Moreover, our objective is to expand the *DSaaS* platform to allow not only physicians but also researchers from different fields to use the tool on a variety of databases.

Future work will enrich the platform with a dataflow editor and unsupervised Machine Learning methods based on topological data analysis. The combination of topological and Machine Learning methods will support the analysis of large datasets [33]. Thus, the *DSaaS* platform will allow users to carry out a complete data analysis pipeline for discovering new patterns, define new models and understanding the trends of data under consideration [34].

## Data Availability

The Dataset used can be provided by the Corresponding Author on reasonable request.

## Abbreviations

ACC: Accuracy
AUC-PRC: Area under Precision-Recall curve
AUC-ROC: Area under receiver operating characteristic curve
CDC: Centers for Disease Control and Prevention
*DSaaS*: *Data Science as a Service*
EBM: Evidence Based Medicine
GA: Genetic Algorithm optimization
FN: False negatives
FP: False positives
FPR: False positive rate
MCC: Matthews correlation coefficient
MDR: Multidrug resistant
MDR UTI: Multidrug resistant urinary tract infection
NN: Neural Networks
REST: Representational state transfer
SEN: Sensitivity
SVM: Support Vector Machine
TN: True negatives
TP: True positives
TPR: True positive rate

## References

[1] Mancini A, Pucciarelli S, Lombardi FE, Barocci S, Pauri P, Lodolini S (2019). Differences between community- and hospital-acquired urinary tract infections in a tertiary care hospital. New Microbiol.

[2] Tlachac ML, Rundensteiner E, Barton K, Troppy S, Beaulac K, Doron S (2018). Predicting future antibiotic susceptibility using regression-based methods on longitudinal Massachusetts Antibiogram data. Biostec.

[3] Barlam TF, Cosgrove SE, Abbo LM, Macdougall C, Schuetz AN, Septimus EJ (2016). Implementing an antibiotic stewardship program: guidelines by the Infectious Diseases Society of America and the Society for Healthcare Epidemiology of America. Clin Infect Dis.

[4] Naber KG, Bergman B, Bishop MC, Bjerklund-Johansen TE, Botto H, Lobel B (2015). EAU guidelines for the management of urinary and male genital tract infections. Urinary tract infection [UTI] working Group of the Health Care Office [HCO] of the European Association of Urology [EAU]. Eur Urol.

[5] Maki DG, Tambyah PA (2001). Engineering out the risk for infection with urinary catheters. Emerg Infect Dis.

[6] Foxman B (2010). The epidemiology of urinary tract infection. Nat Rev Urol.

[7] Woodford HJ, George J (2011). Diagnosis and management of urinary infections in older people. Clin Med J R Coll Phys London.

[8] Lateef F (2009). Hospital design for better infection control. J Emerg Trauma Shock.

[9] Ventola CL (2015). The antibiotic resistance crisis: part 1: causes and threats. P T A Peer-Rev J Formul Manag.

[10] Chawla NV, Bowyer KW, Hall LO, Kegelmeyer WP (2002). SMOTE: synthetic minority over-sampling technique. J Artif Intell Res.

[11] Vapnik VN (1999). An overview of statistical learning theory. IEEE Trans Neural Netw.

[12] Dorogush AV, Ershov V, Gulin A (2018). CatBoost: gradient boosting with categorical features support.

[13] Haykin S (1994). Neural networks: a comprehensive foundation. Knowl Eng Rev.

[14] De Leone R, Capparuccia R, Merelli E (1998). A successive overrelaxation backpropagation algorithm for neural-network training. IEEE Trans Neural Netw.

[15] Kuhn M (2008). Building predictive models in R using the caret package. J Stat Softw.

[16] Rodriguez A (2008). Restful web services: the basics. Online artic IBM dev tech Libr.

[17] Peng G, Ritchey NA, Casey KS, Kearns EJ, Privette JL, Saunders D, et al. Scientific stewardship in the open data and big data era - roles and responsibilities of stewards and other major product stakeholders. D-Lib Mag. 2016;22.

[18] CDC, NHSN (2014). CDC / NHSN surveillance definitions for specific types of infections. Surveill Defin.

[19] Siegel JD, Rhinehart E, Jackson M, Chiarello L (2007). Management of multidrug-resistant organisms in health care settings, 2006. Am J Infect Control.

[20] Flores-Mireles AL, Walker JN, Caparon M, Hultgren SJ (2015). Urinary tract infections: epidemiology, mechanisms of infection and treatment options. Nat Rev Microbiol.

[21] Scrucca L (2015). GA : a package for genetic algorithms in R. J Stat Softw.

[22] Little MA, Varoquaux G, Saeb S, Lonini L, Jayaraman A, Mohr DC (2017). Using and understanding cross-validation strategies. Perspectives on Saeb et al Gigascience.

[23] Kuhn M, Johnson K (2013). Applied predictive modeling. Applied predictive modeling.

[24] Bartocci E, Cacciagrano D, Cannata N, Corradini F, Merelli E, Milanesi L (2007). An agent-based multilayer architecture for bioinformatics grids. IEEE Transact Nanobiosci.

[25] Piangerelli M, Rucco M, Tesei L, Merelli E (2018). Topological classifier for detecting the emergence of epileptic seizures. BMC Res Notes.

[26] Piangerelli M, Maestri S, Merelli E (2020). Visualizing 2-simplex formation of metabolic reactions. Submitted to JMGM.

[27] Mancini A, Eyassu F, Conway M, Occhipinti A, Liò P, Angione C (2018). CiliateGEM: an open-project and a tool for predictions of ciliate metabolic variations and experimental condition design. BMC Bioinformatics.

[28] Alanazi HO, Abdullah AH, Qureshi KN (2017). A critical review for developing accurate and dynamic predictive models using machine learning methods in medicine and health care. J Med Syst.

[29] Bhandari M, Giannoudis PV (2006). Evidence-based medicine: what it is and what it is not. Injury.

[30] Scott IA (2018). Machine learning and evidence-based medicine. Ann Intern Med.

[31] Takaya S, Rehmsmeier M (2015). The precision-recall plot is more informative than the ROC plot when evaluating binary classifiers on imbalanced datasets. PLoS One.

[32] Chicco D, Jurman G (2020). The advantages of the Matthews correlation coefficient [MCC] over F1 score and accuracy in binary classification evaluation. BMC Genomics.

[33] Austenfeld M (2012). A graphical user Interface for R in a rich client platform for ecological modeling. J Stat Softw.

[34] Zou H, Li G (2010). Diagnosis, prevention, and treatment of catheter-associated urinary tract infection in adults: 2009 international clinical practice guidelines from the Infectious Diseases Society of America. Chin J Infect Chemother.

